# Material Basis of the Difference between Hedysari Radix and Honey-Processed Hedysari Radix in Buzhong Yiqi

**DOI:** 10.1155/2020/4543761

**Published:** 2020-12-02

**Authors:** Jiang-tao Niu, Rui Cao, Xin-lei Si, Tian-tian Bian, Er-dan Xin, Yue-feng Li, Xing-ke Yan

**Affiliations:** ^1^Pharmacy of College, Gansu University of Chinese Medicine, Lanzhou 730000, China; ^2^Key Laboratory of Quality and Standard of TCM of Gansu Province, Lanzhou 730000, China; ^3^Acupuncture of College, Gansu University of Chinese Medicine, Lanzhou 730000, China

## Abstract

**Objective:**

To compare the changes of chemical components of Hedysari Radix (HR) before and after honey-processing, and to explore the material basis of the difference between HR and honey-processed Hedysari Radix (HPHR) in Buzhong Yiqi.

**Methods:**

Different compounds in aqueous extracts of HR and HPHR were analysed by UPLC-MS. A rat model of spleen qi deficiency was established. The rats were treated with different doses of water extracts of HR or HPHR, and pathological differences in spleen tissue, serum levels of *D*-xylose, gastrin (GAS) and amylase (AMS) interleukin-2 (IL)-2 and tumour necrosis factor-*α* (TNF-*α*), as well as spleen and thymus indices, were used as indicators. Differences in the efficacy of HR and HPHR in Buzhong Yiqi were studied.

**Results:**

The research showed that compared with the blank group, the spleen tissue of rats in the model group showed spleen tissue damage, which mainly manifested as unclear boundaries between red pulp and white pulp, irregular spleen morphology and irregular arrangement, and the structure of white pulp destruction, less lymphocytes, the number of germinal centers decreased or atrophied. Compared with the model group, the middle and high dose groups of HR and HPHR had protective effects on spleen tissue of spleen-qi deficiency rats, and HPHR had a stronger effect; compared with those in the model group, rats in each treatment group showed remarkably higher serum *D*-xylose, GAS and AMS levels and thymus and spleen indices, and remarkably lower serum IL-2 and TNF-*α* levels, among which HPHR group showed better regulation effect than HR group. A total of 16 differential compounds were found in the aqueous extracts of HR and HPHR, of which 10 compounds in HPHR were up regulated, while 6 compounds were down regulated compare to HR.

**Conclusion:**

The results indicated that both HR and HPHR can improve spleen qi deficiency syndrome of rats, the pharmacodynamic effect of the latter was better than the former. Differences in components of HR and HPHR potentially leading to variations in efficacy.

## 1. Introduction

Hedysari Radix (HR) is the dried root of the leguminous *Hedysarum polybotrys* Hand.-Mazz., which has been extensively used as a traditional Chinese medicine (TCM) for thousands of years. It is the same as Astragali Radix in clinical application [1]. HR is specified to Qi-tonifying (vital energy), strengthen the exterior, induce diuresis to alleviate edema, and promote pus discharge and tissue regeneration [2]. Honey-processed Hedysari Radix (HPHR) is the honey-processed form of HR in TCM, which exhibits effects of tonifying middle and replenishing qi, and is commonly used for the treatment of fatigue, poor appetite and diarrhoea [2]. Buzhong Yiqi, one term of TCM treatment principle, refer to treat spleen qi deficiency by improving spleen function. The spleen is an organ involved in multiple systems and functions in digestion, absorption and immunity [3]. Therefore, spleen qi deficiency syndrome generally shows two kinds of pathological changes; one is the weakening of the spleen transport function [4], and the other is the weakening of the immunity. Processing herbal medicine is a classic characters of TCM clinic. The TCM theory holds that processing with honey can regulate spleen and stomach functions, achieve in reinforcing “Qi” (vital energy) and enhance the effect of medicinal herbs on Buzhong Yiqi [5]. Thus, the efficacy and applications of HR can be expected to change before and after processing with honey. However, it is not clear which stronger about the function of Buzhong Yiqi in HR and HPHR and the difference of their chemical components.

To study differences in the effect of Buzhong Yiqi before and after processing of HR with honey and reveal whether differences in efficacy are related to differences in chemical composition, we first establish a rat model of spleen qi deficiency and then use pathological differences in spleen tissue, thymus and spleen indices and serum interleukin (IL)-2, tumour necrosis factor-*α* (TNF-*α*), *D*-xylose, gastrin (GAS) and amylase (AMS) levels as indicators of treatment effects. UPLC-Q/TOF-MS technology is used to analyse differences in the chemical compositions of HR before and after honey processing. Finally, the material basis of differences in the efficacy of HR and HPHR on Buzhong Yiqi are revealed and discussed.

## 2. Materials and Methods

### 2.1. Experimental Herbs

Samples of HR were collected from Micang Mountain, Wudu District, Longnan City, Gansu Province, and identified as the dry root of *H. polybotrys* Hand.-Mazz. by Professor Wang Mingwei from the Department of Chinese Medicine Identification, School of Pharmacy, Gansu University of Chinese Medicine.

### 2.2. Experimental Animals

90 six-weeks-old Specific pathogen free (SPF) male Sprague-Dawley rats weighing 200 ± 20 g were provided by Lanzhou Veterinary Research Institute of Chinese Academy of Agricultural Sciences (Animal Certificate No. SCXK (Gan) 2015-0002).

The ambient temperature of the breeding room was controlled to 23 ± 2°C, and its relative humidity was controlled to 45%–50%.

### 2.3. Reagents

Aqueous extracts of HR and HPHR containing 0.54 g·ml^−1^ (low-dose), 1.08 g·ml^−1^ (medium-dose), 1.62 g·ml^−1^ (high dose); aqueous rhubarb extract containing 1 g·ml^−1^ raw drug; D-xylose (Tianjin Kaixin Chemical Industry Co., Ltd., Batch No. 20160302); rat *D*-xylose enzyme-linked immunosorbent kit (WKSUBIO, 201704SU-B30087); rat GAS enzyme-linked immunoassay kit (WKSUBIO, 201704SU-B30839); rat AMS enzyme-linked immunoassay kit (WKSUBIO,201704SU-B30316) rat IL-2 enzyme-linked immunosorbent kit (WKSUBIO, 201705SU-B30208); rat TNF-*α* enzyme-linked immunosorbent kit (WKSUBIO, 201704SU-B31063); acetonitrile (Merck, HPLC grade, Batch No. 1499230-935); ammonium acetate (Sigma, HPLC grade, Batch No. 70221); methanol (Merck, HPLC grade, Lot No. 144282); ammonia (Merck, HPLC grade, lot 105426); honey (Guilin Zhoushi Shunfa Food Co., Ltd.); Neutral gum (Beijing Solibao Technology Co., Ltd., G8590); 4% paraformaldehyde (Beijing Solibao Technology Co., Ltd.)); Ihong dyeing solution (Beijing Solibao Technology Co., Ltd., G1100); Hematoxylin (Beijing Solibao Technology Co., Ltd., G1080).

### 2.4. Instruments and Equipment

Ultra-high performance liquid chromatography (UHPLC; Agilent, Agilent 1290 Infinity LC); chromatographic column (Waters, Acquity UPLC HSS T3 1.8 *μ*m, 2.1 mm × 100 mm); mass spectrometer (AB SCIEX, Triple TOF 5600+); 1/100000 analytical balance (Beijing Sartorius Scientific Instrument Co., Ltd., BT125D); enzyme labelling instrument (Epoch, USA Boteng Instrument Co., Ltd.); ultra-low temperature refrigerator (MDF-U71V, Japan Sanyo Co.); high-speed refrigerated centrifuge (Biofuge Stratos, USA); Embedding machine (Jinhua Kedi Instrument Equipment Co., Ltd., Zhejiang, KD-BM); microtome (Germany, LEICA2016); microscope (Ningbo Shunyu Instrument Co., Ltd., RX50).

### 2.5. Honey-Processed HR

Take a certain amount of HR pieces, put them in a rectangular porcelain plate, add refined honey infiltration for 1 h. Refined honey was diluted with distilled water (20% of amount of honey). Approximately 25 g of refined honey was added to 100 g of HR pieces. The oven temperature was set to 70°C, the baking time was set to 2.5 h, and the HR tablets were laid to a thickness of 3 cm in rectangular porcelain plate. After baking in the oven, the tablets were removed, cooled and set aside.

### 2.6. Comparison of Buzhong Yiqi

#### 2.6.1. Experimental Groups

After 7 days of adaptive feeding, 80 SD male rats were randomly divided into a blank group, model group, HR low-dose group (HRL group), HR medium-dose group (HRM group), HR high-dose group (HRH group), HPHR low-dose group (HPHRL group), HPHR medium-dose group (HPHRM group) and HPHR high-dose group (HPHRH group). Each group included 10 rats.

#### 2.6.2. Model Establishment

Except for the blank group, all other groups used the three-factor composite modelling method of limit food intake, make diarrhoea and fatigue to establish the model of spleen qi deficiency [6]. Modelling was performed for 14 days as follows. The model rats were fasted for a single day and given free access to water. On even-numbered days, 75 g·kg^−1^·bw^−1^ food and unlimited drinking water were given. The model rats were intragastrically administered with 1 g·ml^−1^ rhubarb decoction at a dosage of 20 ml·kg^−1^ every day at 12:00 noon. A fuse weighing 10% of the body mass was attached to the base of the tail, and the rats were placed in a swimming cylinder with a temperature of 25 ± 1°C and a depth of 50 cm to swim to exhaustion, as determined by the rats' nose being submerged under water for 10 s, every day for 15 afternoons [7]. After exhaustion, the rats were collected and dried. Normal-group rats were routinely raised without model conditions.

#### 2.6.3. Drug Intervention

With body surface area method [8] and relevant literatures [9], the dosage of intragastric administration in rats was calculated. After the model was successfully established, every day at 8:00 am, rats in the high-, medium- and low-dose groups were respectively given 16.2, 10.8 and 5.4 g·kg^−1^ HR or HPHRR in a corresponding dose of 10 ml·kg^−1^ aqueous extract by intragastric administration. Rats in the blank and model groups were orally administered an equal dose of distilled water. Once a day for 15 days, excluding the blank group, the other groups continued to apply the modelling conditions.

#### 2.6.4. Sample Collection

Exactly 15 days after extract administration, rats in each group were fasted but given free access to drinking water. At 6:00 pm on the same day, the rats were weighed. On the 16th day after extract administration, rats in each group were given 1 ml·kg^−1^ 5% D-xylose solution. After 1 h of gavage, 5 ml of blood samples was collected from the abdominal aorta into non-anticoagulated vacuum blood collection tubes. The blood samples were allowed to stand at room temperature for 3 min and centrifuged at 4000 rpm for 5 min at 4°C, after which the supernatant was placed in 2 ml EDTA tubes and stored in the refrigerator at −20°C. After blood collection, neck-killed rats, and their spleens and thymus were immediately removed and accurately weighed.

#### 2.6.5. HE Staining of Spleen Tissue

The spleen tissues were precisely weighed and fixed in 4% paraformaldehyde fixative solution for 1 week, then embedded in paraffin, sliced, baked, stained with HE tissue, and observed under an inverted microscope at 200×.

#### 2.6.6. Sample Testing

Serum samples were obtained from the abdominal aorta and assayed for IL-2, TNF-*α*, *D*-xylose and GAS by using an enzyme-linked immunosorbent assay kit according to the manufacturer's instructions.

#### 2.6.7. Ethical Committee Number

This animal experiment satisfied the requirements of the Experimental Animal Ethics Committee and was approved by the Ethics Committee (Committee No. 2019-015).

### 2.7. Chemical Composition Analysis

#### 2.7.1. Preparation of Test Solutions

10 batches of HR and HPHR samples were obtained, crushed, sieved through a 40 mesh sieve and dried at 60°C to a constant weight. Exactly 3.0000 g of sample powders of HR and HPHR were accurately weighed and placed into a 150 ml Erlenmeyer flask. Samples were added with 30 ml of distilled water and refluxed for 1 h. Thereafter, the mixture was filtered and extracted three times in succession. The filtrate was combined and concentrated under reduced pressure. Constant volume to 10 ml volumetric flask, numbering, spare [9].

#### 2.7.2. UPLC Chromatographic Conditions

Samples were placed in a 4°C auto sampler and separated on an Acquity HSS T3 (2.1 mm × 100 mm, 1.8 *μ*m) column using an Agilent 1290 Infinity LC UHPC system. The following parameters were adopted: injection volume: 2 *μ*l; column temperature: 25°C; flow rate: 0.3 ml·min^−1^; mobile phase *A*: water + 25 mM ammonium acetate + 25 mM ammonia and mobile phase *B*: acetonitrile. The gradient elution procedure is shown in Table 1. QC samples were inserted into the sample queue to monitor and evaluate the stability of the system and ensure the reliability of the experimental data.

#### 2.7.3. Mass Spectrometry Conditions

Each sample was tested in positive- and negative-ion mode using electrospray ionisation (ESI). Samples were separated and analysed using UPLC and mass spectrometry (Triple TOF 5600+). The ESI source conditions were as follows: ion source gas 1 (Gas1): 60, ion source gas 2 (Gas2): 60, ion source gas (CuR): 30, source temperature: 600°C, ion voltage floating (ISVF): ±5500 V (positive- and negative-ion mode), *m/z* range for ion source current scanning: 60–1200 Da, and *m/z* range for product ion scanning: 25–1200 Da. The cumulative scan time was 0.15 s/spectrum, and the cumulative product ion scan time was 0.03 s/spectrum. Secondary mass spectrometric information was obtained using IDA in high-sensitivity mode with the following parameters: decay potential: ±60 V (positive- and negative-ion mode) and collision energy: 30 eV. IDA is set as follows: Isotope is excluded in 4 Da, candidate ions are monitored every cycle: 6.

### 2.8. Data Processing

The raw data were converted to .mzXML format by using ProteoWizard; XCMS software was then used for peak alignment, retention time correction and peak area extraction. Chemical composition structure identification was achieved by accurate mass matching (<25 ppm) and secondary spectral matching to retrieve Massbank (http://www.massbank.jp) and laboratory self-built databases.

### 2.9. Statistical Methods

If the data showed a normal distribution and their variance was uniform, the results are expressed as mean ± standard deviation ($\bar{x}\pm s$), and comparisons between groups were conducted by using one-way ANOVA. If the variance was not uniform and the data did not have a normal distribution, the W-H rank sum test was used. All of the data were statistically analysed using SPSS 19.0 software. *P* < 0.05 was considered to indicate statistically significant differences, and *P* < 0.01 was considered to indicate highly statistically significant differences.

## 3. Results

### 3.1. Effect on Histopathology of Rat Spleen

**Figure 1:**
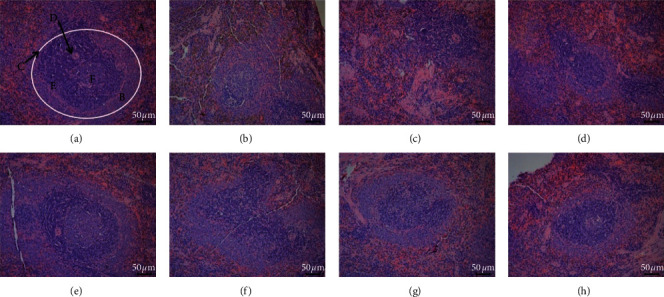
Spleen histological sections of rats in each group (200x). Note: (a) Blank group; (b) Model group; (c) HRL group; (d) HRM group; (e) HRH group; (f) HPHRL group; (g) HPHRM group; (h) HPHRH group. (A) Red pulp; (B) White pulp; (C) The boundary; (D) Germinal center; (E) Lymphocytes; (F) Splenic corpuscles.

The spleen is mainly composed of white pulp, red pulp and marginal area. According to the histopathological results of the spleen of each group of rats in Figure 1, the spleen structure of the blank group is clear, the red and white pulp are neatly distributed, the boundaries are clear, and the lymphocytes are tightly arranged. Compared with the blank group, the red and white pulp of the spleen of the model group the boundaries are not clear, the spleen bodies are irregular and irregularly arranged, the structure of the white pulp is destroyed, less lymphocytes and the number of germinal center is reduced or atrophied. Compared with the model group, the middle and high dose groups of HR and HPHR have protective effects on spleen tissue of spleen-qi deficiency rats, and HPHR has a stronger effect.

### 3.2. D-Xylose and GAS Test Results

As shown in Table 2, compared with the blank group, the model group showed significantly lower serum *D*-xylose, GAS and AMS activity levels (*P* < 0.01). Compared with those in the model group, rats in each of the treatment groups, showed different degrees of increased serum D-xylose, GAS and AMS activity levels (*P* < 0.01or *P* < 0.05). In addition, compared with rats in the HRM group, those in the HPHRM group showed significantly increased serum D-xylose, GAS and AMS activity levels (*P* < 0.01 and *P* < 0.05).

### 3.3. Results of Visceral Index Statistics

Table 3 results showed that, the thymus and spleen indices of the model group are significantly lower than those of the blank group (*P* < 0.01). Compared with those of the model group, rats in each of the treatment groups, except for those of the HRL group, showed significant increases in thymus index (*P* < 0.01); the spleen index of all other treatment groups also showed significant increases (*P* < 0.01 or *P* < 0.05). Compared with those of the HRM group, rats in the HPHRM group showed significantly increased thymus and spleen indices (*P* < 0.01 and *P* < 0.05).

### 3.4. IL-2 and TNF-*α* Test Results

The results in Table 4 show that serum IL-2 and TNF-*α* levels in the model group were significantly higher than those in the blank group (*P* < 0.01). Compared with the model group, the treatment groups showed different degrees of decreased serum IL-2 and TNF-*α* (*P* < 0.05 or *P* < 0.01). Compared with those in the HRM group, rats in the HPHRM group showed significantly lower serum IL-2 and TNF-*α* levels (*P* < 0.01 and *P* < 0.05).

### 3.5. Sample Quality Control Analysis

UPLC/Q-TOF-MS was applied for analysis. Quality control samples (QC) are prepared from a mixture of sample extracts and are used to analyse the repeatability of samples under the same processing method. QC samples were inserted into the sample queue to monitor and evaluate the stability of the system and ensure the reliability of the experimental data. By performing overlapping display analysis on the total ion chromatogram (TIC) of different quality control QC samples mass spectrometry detection analysis, the repeatability of sample extraction and detection can be judged and used to evaluate the stability of the entire system.

**Figure 2:**
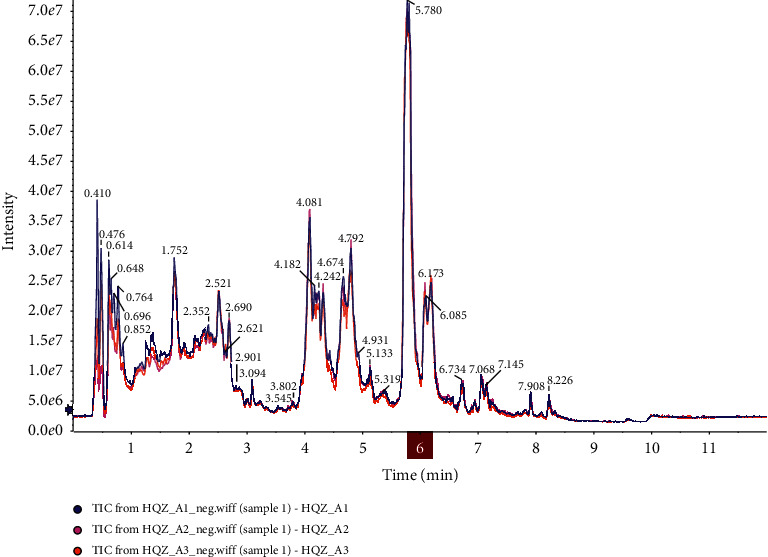
Overlap of QC TIC spectrum detection.

The results in Figure 2 show that the QC sample detection curve has a high degree of overlap, that is, the retention time and peak intensity are consistent, indicating that the mass spectrometry has better signal stability when the same sample is detected at different times. This results indicated that methods were suitable for subsequent sample analysis.

### 3.6. Multivariate Statistical Analysis of Differential Compounds from Aqueous Extracts of HR and HPHR

**Figure 3:**
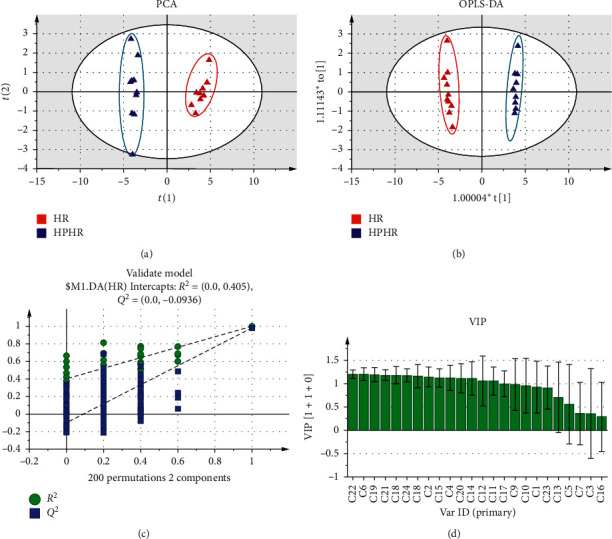
Multivariate statistical analysis of HPHR and HR differential compounds. Note: (a) PCA score plot of the HR and HPHR; (b) OPLS-DA score plot of the HR and HPHR; (c) The 200 times permutation test of OPLS-DA scores of the HR and HPHR; (d) VIP value of the differential chemical compositions of HR and HPHR.

Based on the signal intensity of the compound, we statistically analysed and screened the differential compounds between the aqueous extract of HR and HPHR. We refer to statistically significccant as *P* < 0.05. 24 compounds were selected from the aqueous extract of HR and HPHR. Multivariate statistical analysis of the above-mentioned differential compounds was performed using SIMCA 14.1 software. The results are shown in Figure 3.

As a multivariate statistical analysis method, Principal component analysis (PCA) change many variables into fewer important variables by reducing the dimensionality of datasets. The PCA scores of HR and HPHR (Figure 3(a)) showed that these two groups were separated completely. Orthogonal partial least squares-discriminant analysis (OPLS-DA) focuses on grouping and the differences between grouped samples and has better classification and regression prediction ability. Therefore, the OPLS-DA method was used for further investigation of the difference between HR and HPHR. The OPLS-DA scores of HR and HPHR (Figure 3(b)) showed that both the HR and HPHR group exhibited obvious aggregation, which showing significant difference between two groups. The values of *R*^2^ and *Q*^2^ were 0.9875 and 0.9843 indicating better model-fitting degree, respectively. The permutation test was conducted to check the overfiting of OPLS-DA models. As shown in Figure 3(c), *Q*^2^ intercept < 0, and the intersection of *R*^2^ and *Q*^2^ with the abscissa were less than 0.5, and the intersection of the two with the ordinate was less than 1, indicating the OPLS-DA models were effective without over fitting. The above results showed that there was a significant difference between the HR and HPHR group.

### 3.7. Identification of Chemical Constituents in the Aqueous Extracts of HR and HPHR 

**Figure 4:**
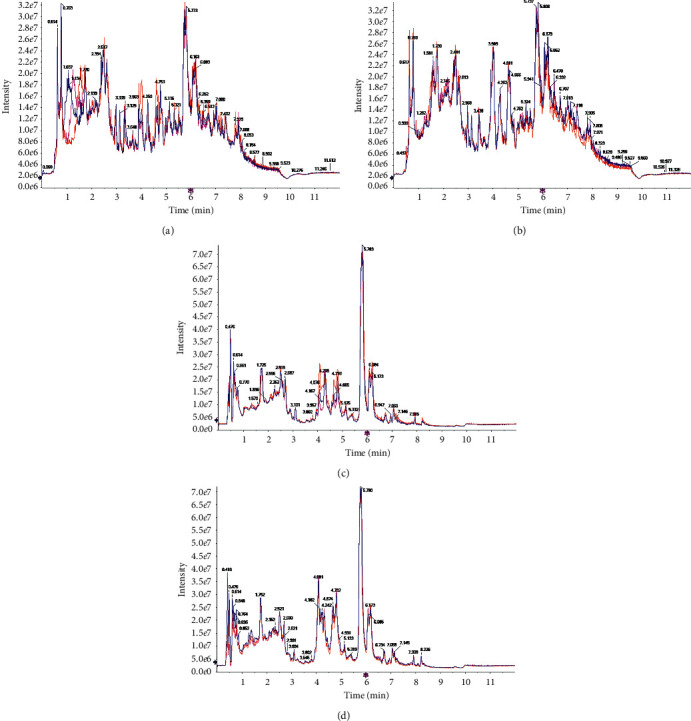
TIC of HR and HPHR water extracts in positive and negative ion mode. Note: (a) total ion current pattern of positive ion mode HR water extract; (b) total ion current pattern of positive ion mode HPHR water extract; (c) total ion current diagram of negative ion mode HR water extract; (d) total ion current diagram of negative ion mode HPHR water extract.

**Figure 5:**
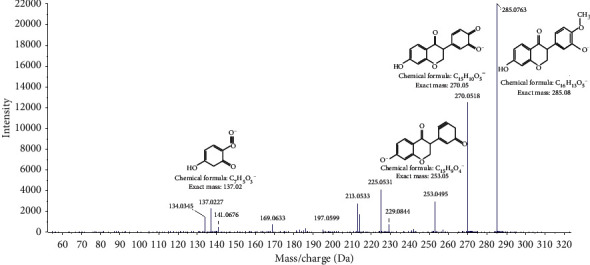
The MS^2^ spectrum in positive mode of calycosin

**Figure 6:**
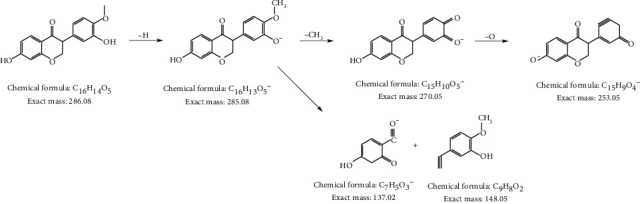
Calycosin cracking method diagram.

Figure 4 showed total ion current of HR and HPHR obtained in positive and negative ion mode. To illustrate the identification process of different compounds, took *m/z* 286.0801 ion at 37.186 seconds in Positive ion mode as an example. The MS^2^ spectrum of *m/z* 286.0801 ion was shown in Figure 5. The possible elemental compositions C_16_H_12_O_5_ of the compound were calculated based on *m/z* value and compared with reference compounds across databases. And then the fragment ions *m/z* 285.0801, *m/z* 270.0512, *m/z* 253.0512, *m/z* 137.02 and their corresponding intensities were compared with those of reference compounds registered in the database. Based on the above process information, the compound was identified as calycosin and its cleavage method is shown in Figure 6. The other compounds were identified as this process method depending on the elemental composition in searching databases. The different compounds from aqueous extracts of HR and HPHR were shown in Table 5.

The results as shown in Table 5 were obtained by the identification and analysis of the mass spectrometry information obtained in the positive and negative ion modes. The results in Table 5 show 24 compounds that could be identified in aqueous extracts of HR and HPHR.

Variable importance in the projection (VIP) value was used to evaluate the potential differential compounds, which were selected based on VIP>1 and statistical significance *P* < 0.05. Based on the results in Figure 3(d) and Table 5, a total of 16 differential components were selected from the aqueous extract of HR and HPHR. Compared with those in HR, 10 compounds were upregulated in HPHR, namely, maltotriose, *D*-mannose, guanosine, cytosine, salbutamol, tripterine, luteolin, betaine, calycosin, and formononetin. In addition, 6 compounds were downregulated in HPHR compared with those in HR, namely, *L*-arabinose, astragalin, adenosine, oxidised linalool, glycitein and apigenin.

## 4. Discussion

According to traditional Chinese medicine theory, spleen qi deficiency includes two aspects, namely, qi deficiency and spleen deficiency. Qi deficiency leads to insufficient defensive qi, which, in turn, reduces the resistance of the body to the invasion of external evils and causes disease. Thus, qi deficiency reduces immune functions. The spleen is the largest immune organ of the human body and plays an important role in regulating the immune functions of the body. When the function of the spleen deteriorates, the immune function of the body is negatively affected [10]. Decreased thymus and spleen indices are manifestations of low immune function [3]. TNF-*α*and IL-2 are cytokines that are closely related to the immune regulation of the body [11]. Serum IL-2 and TNF-*α* levels can reflect the immune function of the body, and levels of IL-2 and TNF-*α* are higher than normal indicates that the body's immune function is disorganised and immunity is reduced [12]. The spleen controls digestion and is closely related to the digestive system. Spleen deficiency syndrome is a comprehensive pathological change characterised by multiple organ and multi-system dysfunction with low digestion and absorptive functions [13]. Spleen deficiency presents as poor absorptive and digestive control, impaired gastrointestinal hormone secretion and gastrointestinal hormone abnormalities [14]. The xylose absorption test is a well-recognised indicator reflecting the digestive and absorptive functions of the spleen and stomach [15]. GAS is a common gastrointestinal peptide hormone that can promote the secretion of gastric acid by parietal cells and growth of mucosal epithelial cells, accelerate the repair of injured gastric mucosal tissue, and participate in the gastric mucosal inflammatory response [16]. GAS also plays an important role in promoting gastric emptying [17]. AMS is a key enzyme for carbohydrate absorption and digestion [18]. AMS activity is a specific indicator that is sensitive to the secretion of digestive enzymes and an important indicator of spleen and stomach digestion function [19].

Deficiency of spleen qi is a TCM symptom. It will impair the normal function of the spleen, and its normal tissue structure will also have pathological changes. According to related studies, the spleen histological sections of rats with spleen qi deficiency showed structural damage, decreased total numbers of lymphocytes in the red pulp area, sparsely distributed and thinner splenic cord in the red pulp area [20, 21]. The results of pharmacodynamic experiments show that, spleen-qi deficiency rats suffered from spleen tissue damage, such as a decrease in lymphocyte count. Lymphocytes are an important cell component of the body's immune response function, and the decrease in their number indicates that the body's immune capacity is weakened. The weakened immunity is one of the pathological changes of spleen-qi deficiency syndrome. The middle and high dose groups of HR and HPHR had protective effects on spleen tissue of Spleen-qi deficiency rats, and the effects of HPHR were slightly stronger.

Compared with those in the blank group, rats in the model group show remarkably decreased thymus and spleen indices, increased serum IL-2 and TNF-*α* levels and decreased *D*-xylose, GAS contents and AMS activity. This finding indicates that the immune function of the model group is weakened, spleen and stomach absorptive and digestive functions are impaired, and spleen deficiency has developed. The model of spleen qi deficiency was successfully established in this study. Compared with those in the model group, rats in each treatment group showed remarkably increased thymus and spleen indices, decreased serum IL-2 and TNF-*α* levels, and increased *D*-xylose, GAS contents and AMS activity; these results suggest that HR and HPHR can promote the recovery of immune functions and enhance the absorptive and digestive functions of the spleen and stomach in rats with spleen qi deficiency. Compared with those in the HRM group, rats in the HPHRM group showed remarkably increased thymus and spleen indices, decreased serum IL-2 and TNF-*α* levels and increased *D*-xylose, GAS contents and AMS activity. These findings suggest that the positive regulatory effect of HPHR on the immunity and spleen and stomach absorptive and digestion functions of spleen qi-deficient rats is stronger than that of HR.

Processing herbal medicine is a classic characters of traditional Chinese medicine (TCM) clinic. The TCM theory holds that the properties or efficacy of herbal medicine can be changed by specific processing methods [22] and processing with honey can regulate spleen and stomach functions, thus to tonify “Qi” (vital energy) and increase the efficacy of Buzhong Yiqi decoction. Accordingly, the results of the above drug efficacy experiments are consistent with the theory of TCM.

Multivariate statistical analysis results showed that there was a significant difference between the HR and HPHR group. Amongst the chemical constituents of aqueous extracts of HR and HPHR, 10 compounds, mainly saccharides, flavonoids, isoflavones and nucleosides, and 6 compounds, mainly flavonoids, were respectively upregulated and downregulated in the latter when compared with those in the former. Xylose and arabinose in saccharide compounds are important components of *H. polybotrys* polysaccharides (HPS) [23, 24], which improve immunity and present anti-oxidation and anti-tumour activities [25]. Indeed, HPS is the main component of HR tonics. Calycosin and formononetin are the main active flavonoids of HR; these compounds possess anti-oxidative, anti-viral, anti-ischaemic, and blood-improving effects [26, 27]. Celastrol possess anti-inflammatory, immunosuppressive and anti-tumour activities [28, 29]. Taken together, the results reveal differences in the components and contents of these components between HR and HPHR.

HPHR came from HR processed with honey with a TCM processing method. The differential compounds between HR and HPHR mainly involve 3 upregulated compounds (saccharides, free flavonoids and free isoflavones) and 1 downregulated compound (flavonoid glycosides). It is suggested that honey processing may break flavonoid glycosides into free flavonoids and saccharides, lead to up regulate free flavonoids and saccharides, and down regulate flavonoid glycosides.

## 5. Conclusion

The above analysis showed that both HR and HPHR can remarkably improve spleen qi deficiency syndrome in rats; however, the pharmacodynamic effect of the latter is better than that of the former. There are different components in HR and HPHR and these differences may be the active substances that lead to the difference of pharmacodynamics between HR and HPHR.

## Figures and Tables

**Table 1:** Gradient elution procedure for UPLC analysis.

Time (min)	*A* (%)	*B* (%)
0	5	95
0.5	5	95
7	45	65
8	60	40
9	60	40
9.1	5	95
12	5	95

**Table 2:** Determination of serum D-xylose, GAS and AMS activity in each group of rats ($\bar{x}\pm s$).

Group	*n*	D-xylose (ng·mL^−1^)	GAS (pg·mL^−1^)	AMS (U·L^−1^)
Blank group	10	4.51 ± 0.32	55.26 ± 4.73	339.65 ± 10.02
Model group	8	3.17 ± 0.22^△△^	34.76 ± 5.26^△△^	211.74 ± 13.37^△△^
HRL group	9	3.32 ± 0.31	37.71 ± 4.76	227.75 ± 8.04^*∗∗*^
HRM group	10	3.50 ± 0.32^*∗∗*^	41.95 ± 5.29^*∗*^	252.71 ± 8.26^*∗∗*^
HRH group	10	3.73 ± 0.28^*∗∗*^	48.44 ± 3.50^*∗∗*^	304.47 ± 9.10^*∗∗*^
HPHRL group	10	3.63 ± 0.28^*∗∗*^	43.06 ± 5.30^*∗*^	273.88 ± 8.43^*∗∗*^
HPHRM group	10	3.90 ± 0.30^*∗∗*^^##^	49.79 ± 4.60^*∗∗*^^#^	323.40 ± 3.88^*∗∗*^^##^
HPHRH group	10	3.91 ± 0.29^*∗∗*^	51.94 ± 3.50^*∗∗*^	326.49 ± 7.47^*∗∗*^

Note: ^△△^indicates *P* < 0.01 compared with the blank group;^*∗*^indicates *P* < 0.05 compared with the model group;^*∗∗*^indicates *P* < 0.01 compared with the model group;^#^indicates *P* < 0.05 compared with the HRM group; ^##^indicates *P* < 0.01 compared with the HRM group.

**Table 3:** Results of thymus index and spleen index of rats in each group ($\bar{x}\pm s$).

Group	*n*	Thymus index (mg·g^−1^)	Spleen index (mg·g^−1^)
Blank group	10	0.94 ± 0.06	1.85 ± 0.06
Model group	9	0.67 ± 0.06^△△^	1.61 ± 0.04^△△^
HRL group	9	0.73 ± 0.05^*∗∗*^	1.68 ± 0.05
HRM group	10	0.79 ± 0.06^*∗∗*^	1.74 ± 0.04^*∗∗*^
HRH group	10	0.83 ± 0.03^*∗∗*^	1.80 ± 0.05^*∗∗*^
HPHRL group	10	0.76 ± 0.04^*∗∗*^	1.73 ± 0.03^*∗*^
HPHRM group	10	0.89 ± 0.04^*∗∗*^^##^	1.83 ± 0.05^*∗∗*^^#^
HPHRH group	10	0.88 ± 0.02^*∗∗*^	1.82 ± 0.06^*∗∗*^

Note: ^△△^indicates *P* < 0.01 compared with the blank group; ^∗^indicates *P* < 0.05 compared with the model group; ^∗∗^indicates *P* < 0.01 compared with the model group;^#^indicates that compared with the HRM group *P* < 0.05;^##^indicates *P* < 0.01 compared with the HRM group.

**Table 4:** Determination of serum IL-2 and TNF-*α* levels in rats of each group ($\bar{x}\pm s$).

Group	*n*	IL-2 (pg·*μ*L^−1^)	TNF-*α* (pg·*μ*L^−1^)
Blank group	10	203.69 ± 8.06	41.52 ± 2.09
Model group	8	276.64 ± 10.29^△△^	52.01 ± 2.19^△△^
HRL group	9	259.86 ± 11.17^*∗*^	49.70 ± 2.39
HRM group	10	245.32 ± 4.73^*∗∗*^	46.98 ± 3.03^*∗*^
HRH group	10	232.60 ± 12.91^*∗∗*^	45.36 ± 2.78^*∗∗*^
HPHRL group	10	244.33 ± 10.79^*∗∗*^	47.45 ± 2.48^*∗*^
HPHRM group	10	229.47 ± 7.20^*∗∗*^^##^	44.25 ± 0.84^*∗∗*^^#^
HPHRH group	10	228.29 ± 6.36^*∗∗*^	44.20 ± 1.03^*∗∗*^

Note: ^△△^indicates *P* < 0.01 compared with the blank group;^∗^indicates *P* < 0.05 compared with the model group;^∗∗^indicates *P* < 0.01 compared with the model group;^#^indicates *P* < 0.05 compared with the HRM group;^##^indicates *P* < 0.01 compared with the HRM group.

**Table 5:** Different compounds identified from aqueous extracts of HR and HPHR.

Number	Additive ion	*m/z*	*Rt* (s)	Chemical formula	Chemical compound	Content change	Classification
1	(M + CH_3_COO)-	401.1289	495.214	C_12_H_22_O_11_	Isomaltose	↑^*∗∗*^	Saccharides
2	M+	504.1686	266.273	C_18_H_32_O_16_	Maltotriose	↑^*∗∗*^	Saccharides
3	(M + CH_3_CN + H)+	192.0879	163.902	C_5_H_10_O_5_	D-xylose	↑^*∗*^	Saccharides
4	(2M + H)+	301.1060	34.252	C_5_H_10_O_5_	L-arabinose	↓^*∗∗*^	Saccharides
5	(M + NH_4_)+	360.1505	346.728	C_12_H_22_O_11_	Sucrose	↑^*∗*^	Saccharides
6	(M + H-H_2_O)+	163.0600	239.502	C_6_H_12_O_6_	D-Mannose	↑^*∗*^	Saccharides
7	(M + CH_3_CN + H)+	206.1020	128.180	C_6_H_12_O_5_	L-fucose	↑^*∗*^	Saccharides
8	M+	448.1064	373.428	C_21_H_20_O_11_	Astragalin	↓^*∗*^	Glycosides
9	(M-H_2_O-H)-	248.0783	93.979	C_10_H_13_N_5_O_4_	Adenosine	↓^*∗*^	Nucleoside
10	(M + H)+	417.1180	159.152	C_21_H_20_O_9_	Daidzin	↑^*∗∗*^	Isoflavone
11	(M + H)+	284.0921	184.435	C_10_H_13_N_5_O_5_	Guanosine	↑^*∗*^	Nucleoside
12	(2M-H)-	221.0808	55.663	C_4_H_5_N_3_O	Cytosine	↑^*∗*^	Nucleoside
13	(M-H)-	111.0206	84.037	C_4_H_4_N_2_O_2_	Uracil	↑^*∗*^	Nucleoside
14	M+	239.1486	58.902	C_13_H_21_NO_3_	Salbutamol	↑^*∗*^	
15	(M + H-H_2_O)+	153.1274	153.176	C_10_H_18_O_2_	Oxidation linalool	↓^*∗∗*^	Ethers
16	M+	265.1120	317.969	C_12_H_18_C_l2_N_4_OS	Thiamine	↓^*∗*^	Vitamin
17	(M-2H + 3Na)+	517.2392	381.388	C_29_H_38_O_4_	Celastrol	↑^*∗∗*^	Triterpenoids
18	(M + NH_4_)+	304.0875	191.723	C_15_H_10_O_6_	Luteolin	↑^*∗*^	Flavonoids
19	(M + H)+	285.0759	159.472	C_16_H_12_O_5_	Glycitein	↓^*∗*^	Isoflavone
20	(M + H)+	271.0602	39.835	C_15_H_10_O_5_	Apigenin	↓^*∗*^	Flavonoids
21	(M + H-H_2_O)+	100.0764	48.831	C_5_H_11_NO_2_	Betaine	↑^*∗*^	Alkaloids
22	(M + H)+	286.0801	37.186	C_16_H_12_O_5_	Calycosin	↑^*∗∗*^	Isoflavone
23	(M + H)+	553.1491	42.693	C_22_H_22_O_10_	Calycosin-7-O-beta-D-glucoside	↑^*∗*^	Isoflavone
24	(M + H)+	269.0807	186.693	C_16_H_12_O_4_	Formononetin	↑^*∗*^	Isoflavone

Note: ^*∗*^indicates *P* < 0.05 compared with the HR group, ^*∗∗*^indicates *P* < 0.01 compared with the HR group.

## Data Availability

The data used to support the findings of this study are available from the corresponding author upon request.
